# Methodological approaches in application of synthetic lethality screening towards anticancer therapy

**DOI:** 10.1038/sj.bjc.6605000

**Published:** 2009-03-24

**Authors:** D Canaani

**Affiliations:** 1 Department of Biochemistry, Faculty of Life Sciences, Tel Aviv University, Ramat Aviv, Tel Aviv 69978, Israel

**Keywords:** chemical synthetic lethality, chemical genetic interactions, genetic synthetic lethality, oncogenes, tumour suppressor genes, RNAi

## Abstract

A promising direction in the development of selective less toxic cancer drugs is the usage of synthetic lethality concept. The availability of large-scale synthetic low-molecular-weight chemical libraries has allowed HTS for compounds synergistic lethal with defined human cancer aberrations in activated oncogenes or tumour suppressor genes. The search for synthetic lethal chemicals in human/mouse tumour cells is greatly aided by a prior knowledge of relevant signalling and DNA repair pathways, allowing for educated guesses on the preferred potential therapeutic targets. The recent generation of human/rodents genome-wide siRNAs, and shRNA-expressing libraries, should further advance this more focused approach to cancer drug discovery.

Advances in our understanding of the molecular basis of cancer initiation, progression and metastatic expansion have raised hopes for quick translation of this new data for improved cancer detection, classification, monitoring and especially treatment. However, as pointed out by Varmus (2006), despite the significant progress in several individual cancers and the recent annual 1% decline in cancer-caused mortality rate in the United States, after several decades of steady increases, there are still major obstacles and challenges. The complexity of the problem is also manifested by the low success rate (for 1991–2000) in oncology drugs entering the clinical phase (5%), as compared with the 11% average rate of success for all therapeutic areas (Kola and Landis, 2004). Unfortunately, in terms of costs, the vast majority of drug attrition in all disease areas is at late stages of clinical drug development (phases II and III). In 2000, the major causes of attrition in the clinic had been lack of sufficient efficacy (30%) and toxicity (30%) pertaining to both off-target and on-target toxicity (Kola and Landis, 2004).

Two recent reviews have dealt with the formidable problem of identification of drugs with improved efficiency and cancer selectivity (Benson *et al*, 2006; Collins and Workman, 2006). The former review by a Novartis Corp. group defined four tracks of cancer targets: genetics, synergy, lineage and host. These four subtypes of cancer targets can be viewed as ‘dependencies’, which constitute potential weaknesses – Achilles heels that are unique to cancer cells and can thus be exploited for therapy. The synergy track constitutes synthetic lethal genetic interactions.

The purpose of this minireview is to examine the methodologies that dictate the experimental plans and progress, in application of the concept of synthetic lethality for the identification of lead compounds/drugs, and to a limited extent also of targets, for cancer therapy. Noteworthy is the extensive review by Kaelin (2005) on ‘The concept of synthetic lethality in the context of anticancer therapy’, which presents the issue.

## The basics of synthetic lethality

**Figure 1:**
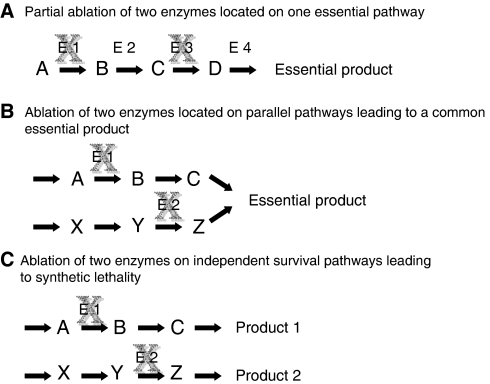
(**A**) Partial ablation of two enzymes located on one essential pathway. (**B**) Ablation of two enzymes located on parallel pathways leading to a common essential product. (**C**) Ablation of two enzymes on independent survival pathways leading to synthetic lethality. Three modes of cell survival pathways amenable for analysis by the synthetic lethality screening approach.

Synthetic lethality describes a cellular condition in which two (or more) non-allelic and non-essential mutations, which are not lethal on their own, become deadly when present within the same cell. The synthetic lethal mutations may constitute partial mutations present together in a single linear essential pathway (Figure 1A), reside in parallel pathways leading to the synthesis of a common essential gene product (Figure 1B) or constitute independent parallel survival pathways each serving as salvage pathway in the absence of the other (see Figure 1C). Intermediate situations in which two mutant genes may generate a ‘synthetic sickness’ condition might also exist. These occurrences may become lethal and lose viability when combined with one or more additional non-essential mutation/s.

Although tentatively, the two mutant synthetic lethal genes are presumed to represent loss-of-function mutations, a condition of synthetic lethality between an overexpressed ‘gene of interest’ and a mutant null gene should also be taken into account (see below) and has been initially described in yeast as a ‘synthetic dosage lethality’ phenotype (Kroll *et al*, 1996).

The first methodology for genetic synthetic lethality screen has been initially developed in yeast, *Saccharomyces cerevisiae* (Bender and Pringle, 1991). A wild-type copy of the ‘gene of interest’, on an unstable episomal plasmid (containing an origin of DNA replication but no centromere), is introduced into yeast cells that are null for expression of this gene. Random mutagenesis of the entire yeast genome within these cells may inactivate a gene that is synthetic lethal with deficiency in the gene of interest. Under these conditions, retention of the episomal plasmid, which is otherwise spontaneously lost, and expression of the gene of interest become essential for survival. Plasmid loss or retention is detected by changes in colony pigmentation. Identification of the mutagenised synthetic lethal gene is accomplished by transforming the particular colony cell population with a wild-type yeast genomic library, while selecting for a change in pigment colour reflecting a loss for the need of the episomal plasmid (i.e., loss of the synthetic lethal condition). The availability of numerous yeast knockout mutants, and the implied added advantage of the method in revealing interactions that do not necessarily require physical interaction between the two gene products, has quickly made this method one of the most powerful in yeast genetics.

Two different modifications of the basic concept have created genetic synthetic lethality screens that are less tedious, more informative and thus suitable for genome-wide analysis. In one, termed synthetic genetic array (SGA), double mutants are generated by mating the query mutant haploid strain to a panel of yeast knockout mutant strains; unviable double-mutant meiotic progeny identifies synthetic lethal relationships (Tong and Boone, 2005). The alternative method named dSLAM for diploid-based Synthetic Lethality Analyzed by Microarray (Pan *et al*, 2004) takes advantage of TAGs representing DNA bar codes, which uniquely mark each yeast deletion allele. These TAGs are flanked by shared priming sequences for PCR. The query mutation is introduced into a pool of heterozygote diploid knockout yeast strains by integrative transformation. Following sporulation, the haploid single (mock transfection)- and double-mutant populations are selected. The presence of every individual deletion mutant within any of the two pools is assayed by measuring the relative abundance of its corresponding TAG-containing PCR product in genomic DNA prepared from each one of the pools, and competitively hybridising to a TAG oligonucleotide microarray. The disappearance of a particular TAG from the double-mutant cell population then may point to a synthetic lethal interaction. The addition of the SGA and dSLAM versions of the yeast genetic synthetic lethality screening enables identification of gene networks and cellular pathways that ‘buffer’ each other biologically (Ooi *et al*, 2006).

The first to suggest the usage of synthetic lethality screening, chemical as well as of genetic, for the development of cancer therapy have been Hartwell and Friend (Hartwell *et al*, 1997). However, figuring that the state of genetic manipulations in mammalian and human-cell systems, in particular, was not ripe yet for genome-wide genetic synthetic lethality screening (a situation that has changed dramatically with the introduction of synthetic siRNAs in 2001), they suggested the use of model genetic systems, such as yeast, the nematode *Caenorhabditis elegans* and the *Drosophila melanogaster* fruit fly.

## Chemical synthetic lethality screening in yeast

In their search for cancer-specific genetic changes, which could form potential selective therapeutic targets, Hartwell and Friend relied on the fact that one of the hallmarks of cancer is genetic instability. This instability is primarily caused by defects in DNA repair, in cell cycle checkpoints and in other cell cycle controls. Moreover, because these processes have been well conserved between yeast and humans, these investigators used a panel of up to 70 isogenic strains, each defective in either DNA repair or cell cycle control gene/genes, for the systematic testing of the 33 most common FDA-approved anticancer cytotoxic drugs (Hartwell *et al*, 1997; Simon *et al*, 2000). This screen identified targets which sensitise chemotherapy drugs administered to yeast. The bifunctional alkylating agent cisplatin, for example, was killing 100-fold better (IC_50_ decreased by two logs) in the presence of defects in either the *Rad6* or *Rad18* post-replication repair genes. Similarly, defects in the *Rad50*, *Rad51* or *Rad52* double-strand break repair through homologous recombination (HR) augmented by almost two logs the killing efficiency by topoisomerase inhibitors, such as camptothecin (Topo I) and mitoxantrone (Topo II). Obviously, these chemotherapeutic-sensitising links call to be tested with regard to their human orthologues in normal and tumour-derived cell lines.

Also screened were more than 85 000 compounds from the collection of the Developmental Therapeutics Program (DTP) branch of NCI (Dunstan *et al*, 2002). This large-scale chemical screen (which was part of the Seattle project based at the Fred Hutchinson Cancer Research Center) identified 126 compounds that were selectively toxic to yeast cells defective in double-strand break repair (rad 50/52 specific). Out of these, 87 compounds were known or closely related to known topoisomerase inhibitors, and 39 were of no known function. The manuscript relating to this screen (Dunstan *et al*, 2002) described the analysis of eight such compounds. The rest of the information (updated to 2002) was made available at the DTP website http://www.dtp.nci.nih.gov/yacds/index.html under ‘NCI Yeast Anticancer Drug Screen’. Among the eight compounds analysed, seven were toxic to mammalian cells; five classified as topoisomerase I poisons and two as topoisomerase II inhibitors. This feasibility/proof-of-concept study showed, in part, the pros and cons in using a model organism for the identification of anticancer drugs in humans.

A boost to the usage of chemical synthetic lethality screens in yeast has been achieved by integrating drug-sensitivity profiles with those of the large-scale genetic interaction data obtained through genome-wide genetic synthetic lethality screens performed by either SGA (Tong and Boone, 2005) or dSLAM (Pan *et al*, 2004; Ooi *et al*, 2006). The clustering of the two profiles links compounds to their protein targets and/or target pathways (Parsons *et al*, 2004; Lopez *et al*, 2008).

Recently, the screening of chemical libraries in panels of knockout collections of *S. cerevisiae,* for unravelling chemical synthetic lethality relationships, has been complemented through usage of collections of mutant yeast strains generated by meiotic recombination (Perlstein *et al*, 2006).

## Chemical synthetic lethality in mammalian cells

### Methodology: high-throughput chemical screening

Over the past decade, three general methods of mammalian-cell-based HTS for synthetic lethal compounds have been reported. The first is the classical screening for genotype-specific inhibitors that are differentially toxic to mutant cell lines grown in multiwell plates. For example, the group led by Stuart Schreiber has screened marine sponge extracts for replication inhibitory activity specific for mouse embryo fibroblasts with either p53^−/−^ or p21^kipl−/−^ genotype *vs* wild type. The assay monitored BrdU incorporation by cytoblotting (Stockwell *et al*, 1999). This classical differential toxicity screening method is subject to several well-known disadvantages: errors due to differences in cell density relating to variations in cell-plating efficiency or growth rate; mistakes resulting from differences in microenvironment growth conditions among plates and wells (particularly regarding circumference effects); the inability of most cell viability assays for multiple readings from zero time on; and the high costs of tissue culture ware and time required for growth analysis. Two methods were independently developed to circumvent some of these shortcomings. In one, developed by Kinzler and co-workers (Torrance *et al*, 2001), two isogenic human colon cancer cell lines, each marked by a distinct mutant GFP gene, were co-cultured into the same wells in multiwell plates. The two isogenic cell lines differed in that in one the mutant *K-Ras* oncogene allele was deleted by HR to generate a null allele. The co-culturing of the two isogenic cell lines supplied a highly important internal control and created an ‘even growth environment’ that minimised potential differences in cell density. The tagging of each cell line by a distinct GFP mutant (capable of double-label reading) allowed for multiple time-point assessments of the relative cell viability through GFP fluorescence monitoring. Yet, this method still has two cell lines that may have different growth rates and the co-culturing of which might render one sensitive to growth-enhancing/inhibitory paracrine signals secreted by the other.

The second recently developed method for high-throughput chemical (and genetic) synthetic lethality screening was generated for human cells (Simons *et al*, 2001a) and for mouse embryo fibroblasts (Einav *et al*, 2003), by my research group. In trying to establish a direct method for finding *bona fide* synthetic lethality relationships between a chemical and a mutated gene (or between two mutated genes in the genetic screen), we resorted to the concept of the original equivalent yeast method, that is, complementation expression of a gene of interest through a low-copy-number unstable episomal replication, in which retention of the episome is forced either by a selectable marker or by synthetic lethal pairing with another gene. Under such synthetic lethal conditions, retention of the episomal plasmid, expressing the wild-type gene of interest, becomes indispensable for viability. Tagging of the chromosomal host DNA and the episomal ‘survival plasmid’ with different GFP variants compatible for double-label fluorescence reading allows for the normalised quantitative detection of the episomal plasmid. Microtitre plates were seeded with either *HPRT1*-deficient human HT1080 fibrosarcoma cells or *HPRT1* null mouse embryo fibroblasts as model systems. We have then shown using HPRT1-expressing Epstein–Barr Virus-based plasmid replicons that one can screen and detect synthetic lethal chemicals (Simons *et al*, 2001a; Einav *et al*, 2003) as well as genes (Simons *et al*, 2001b; Einav *et al*, 2005). This purine biosynthesis *de novo* and salvage pathways leading to the synthesis of the common essential product GMP are an example for the model shown in Figure 1B. The major advantage of this system is that it utilises a single cell line that is thus immune to variations in cell growth characteristics of isogenic cell line pairs or their putative paracrine interactions with each other within the same well. Also, the assay's direct proof for a synthetic lethality condition (i.e. retention of the ‘survival plasmid’) spares a lot of controls needed when using the two other methods that are based on the principle of differential toxicity. On the basis of our experience, it is worthy to note that usage of the GFP variant ratio tends to distort results somewhat. At the same time, other viability detection methods, such as respiration rates or ATP pools, may also harbour the disadvantage of non-linearity, particularly in response to increased cell density. It is for this reason that all HTS methodologies need to be well calibrated to ensure linear responsiveness towards all system parameters. Usage of unstable GFPs that have higher turnover rates may potentially improve matters. Yet, in our model system, the expression level of a GFP variant with a half-life time of 4 h was too low for accurate monitoring. Also, while attempting to use episomal systems established in either breast carcinoma-derived cell lines or MEFs, for screening chemical libraries containing very large numbers (thousands) of chemicals, we observed loss of episomes. This is to say that continuous large-scale cell propagation may lead to the loss of the episome, despite selection for a dominant selectable marker gene encoded by the episome. Concomitantly, there is an increased rate of integration into the host chromosome, supporting resistance to the selectable marker. In lieu of the above, the use of the episomal system should be restricted to validation experiments or small-scale experiments in which the episome containing transfectant cell line is limited to a low passage number following its generation.

### Screening for chemicals synthetic lethal with activated oncogenes

Starting from an isogenic pair of mouse mammary epithelial cell lines, one of which is stably expressing an ectopic *neu/Her2* oncogene, Leder and co-workers screened a chemical library containing 16 000 compounds. One molecule, F16, a low-molecular-weight delocalised lipophilic cation, preferentially accumulated in the inner mitochondrial matrix due to its elevated membrane potential (Δψ_m_) (Fantin *et al*, 2002). This accumulation led to the perturbation of mitochondrial homoeostasis and eventual preferential cell death of the neu-overexpressing cells. Further study has shown a linkage between F16 activity and transformed cells displaying increased mitochondrial membrane potential, rather than neu overexpression *per se*. In terms of its potential cancer therapy usage, F16 is still at the discovery stage (Fantin and Leder, 2006).

Another HTS of chemical libraries centred on tumour cells overexpressing the Ras oncogene. Using their human colon cancer isogenic cell lines in which a mutant *K-Ras* allele has been deleted by targeted HR in one of the two cell lines (see above), the groups led by Kinzler and Vogelstein have screened some 30 000 low-molecular-weight compounds (Torrance *et al*, 2001). Several compounds with up to six-fold greater toxicity towards the K-Ras-expressing cell line/s were identified. The cytidine nucleoside analogue was among the compounds showing activity in xenograft mouse models *in vivo*. Stockwell's group has performed two carefully designed large-scale differential toxicity screens for chemicals synthetic lethal with oncogenic *Ras* expressed in human cancer cells. A cell system, engineered by Weinberg's group, in which primary human forskin fibroblasts (BJ cells) were made immortal, transformed and tumourigenic by the constitutive expression of hTERT, SV40 large and small T oncogenes, and activated Ras^V12^ was used. Initially, 23 550 compounds and later 47 725 chemicals were screened for *H-RAS*/*K-RAS*-dependent synthetic lethality (Dolma *et al*, 2003; Yang and Stockwell, 2008, respectively). The first screen identified several known chemicals, as well as a novel compound named erastin, for ‘eradicator of RAS and Small T-expressing cells’, which displayed a modest eight-fold tumour selectivity in this *in vitro* model cell system, while causing a non-apoptotic cell death.

The second screen (Yang and Stockwell, 2008) identified two compounds, named RSL5 and RSL3, for ‘oncogenic-*RAS*-selective/lethal compounds’. These cause, like erastin, a non-apoptotic, *MEK*-dependent and iron-dependent oxidative cell death. It is worth noting that cells transformed by oncogenic RAS have an increased iron content relative to their normal counterparts. Both RSL5 and erastin target VDAC3, a voltage-dependent anion channel 3. Interestingly, RSL3, whose mechanism of action is not clear yet, has been tested on the NCI60 panel of human tumour cell lines, and is found to have a unique sensitivity/resistance profile, as defined by the COMPARE algorithm, relative to the thousands of compounds already tested at NCI (Yang and Stockwell, 2008).

### Synthetic lethality with tumour suppressor gene deficiency

The screening for chemicals synthetic lethal with a tumour suppressor gene deficiency, in isogenic pairs of tumour cell lines, poses a particular problem for the reference/control cell line. The generation of control ‘tumour suppressor gene-corrected cell line’, by restoring the normal function of an aberrant destabilised tumour suppressor protein with a stabilising ligand, is rarely possible (see the case for the p53 tumour suppressor by Boeckler *et al*, 2008). On the other hand, ectopic expression of the wild-type gene in the tumour suppressor-deficient cell line usually leads to cell cycle arrest and/or cell death (see below for the *von Hippel-Lindau* (*VHL*) gene exception). Thus, researchers resorted to the usage of either reconstructed human model systems (such as BJ cells based) or intermediate-stage tumour cells, whose tumour suppressor gene/s product/s can be sequestered by siRNA/shRNA, or tumour suppressor-binding oncogenic proteins (SV40 large T antigen, HPV E6/E7, etc.). Alternatively, investigators have chosen to design their experimental screening systems based on understanding the consequences of particular tumour suppressor gene deficiency.

#### The case for VHL

The *VHL* tumour suppressor gene is inactivated in about 75% of clear-cell renal cell carcinoma (CC-RCC) patients. Screening for chemicals or downregulated genes synthetic lethal with *VHL* inactivation was made possible by the observation by Kaelin's group that unlike most human tumour suppressor genes ectopic expression of VHL does not lead to inhibition of cell growth or of cell-cycle distribution *in vitro*, but rather to suppressing tumour formation in nude mice xenografts (Iliopoulos *et al*, 1995). One function of VHL is as an E3 ubiquitin ligase, which targets the hypoxia-inducible factor *α* (HIF-*α*) transcription factor for oxygen-dependent proteolysis. Using *in silico* analysis of existing publicly available drug profiles of the NCI60 cell lines, chromomycin A3 (ChA3) was identified as being potentially lethal with *VHL* deficiency in CC-RCC. This HIF-dependent inhibition was first confirmed *in vitro*, followed by *in vivo* mouse xenograft validations (Sutphin *et al*, 2007). The same group (of Amato Giaccia) then screened a 64 000 compound library against VHL-deficient *vs* VHL-complemented RCC cells to identify the STF-62247 as a chemical synthetic lethal with *VHL* deficiency *in vitro* (25-fold difference in IC_50_), as well as *in vivo*. Unlike ChA3, STF-62247 cytotoxicity is HIF independent and engages autophagy induction (Turcotte *et al*, 2008). *VHL* deficiency and its synergistic lethal inhibitors represent examples of the model shown in Figure 1A.

#### The case for PTEN

The *PTEN* (phosphatase and tensin homologue deleted on chromosome 10) tumour suppressor gene is the second most frequently mutated tumour suppressor gene after p53. PTEN antagonises the PI3K/AKT signal transduction pathway by dephosphorylating the PIP_3_ second messenger, which itself is formed from PIP_2_ by PI3K-dependent phosphorylation. In the absence of PTEN, AKT is activated by its recruitment to the cell membrane through its ligand – PIP_3_. The AKT kinase has many downstream substrates involved in apoptosis, cell proliferation and protein synthesis, one of which is mTOR (mammalian target of rapamycin). The synthetic lethality relationship between PTEN deficiency and mTOR-overexpression inhibition was discovered by showing an enhanced sensitivity of PTEN-deficient tumours to the mTOR inhibitor CCI-779, a rapamycin homologue (Neshat *et al*, 2001; Podsypanina *et al*, 2001). Thus, the search for PI3K/AKT/mTOR inhibitors that are synthetic lethal, with PTEN deficiency in particular tumours, was intensified (see recent review by Yap *et al*, 2008). In a complementary elegant approach, the group led by Pamela Silver decided to try restoring at least some of the outcomes of *PTEN* function as a tumour suppressor gene by screening a renal carcinoma cell line (that is deficient in *PTEN*) for chemicals that relocalise the FOXOla transcription factor to the nucleus (Kau *et al*, 2003). AKT is a negative regulator of FOXOla. In *PTEN* null cells, AKT is activated and phosphorylates FOXOla, which is thus mislocalised to the cytoplasm, where it is unable to inhibit cell cycle progression. However, FOXOla is still capable of shuttling between the cytoplasm and the nucleus. Using a FLAG tagging for ectopic FOXOla expressed in the host PTEN-deficient tumour cells and an immunostaining-imaging assay, 18 000 compounds were screened for imposing FOXOla nuclear retention. Forty-two lead compounds were identified, about half of which worked as general nuclear export inhibitors by targeting the CRM1 export transporter. Most of the other chemicals inhibited PI3K/AKT signalling. A large part of these 42 inhibitors blocked cell proliferation in a short-term assay (Kau *et al*, 2003).

#### The case for BRCA1/BRCA2

Our DNA is continually damaged by either endogenous activities, such as DNA replication and cellular free-radical generation, or exposure to environmental agents. This leads to diverse lesions, such as double- or single-strand breaks (DSBs and SSBs, respectively), base modifications and intrastrand or interstrand cross-links. Double-strand breaks are considered to be the most toxic of all DNA lesions. In mammalian cells, DSBs are generally repaired by non-homologous end-joining during G_0_, G_1_ and early S phases of the cell cycle, and by HR during the S–G_2_ phases of the cell cycle. The *BRCA1* and *BRCA2* human tumour suppressor genes are deficient in a subset of breast, ovarian and prostate cancers, and are normally required (among their other roles) for DSB repair by HR. The most common spontaneous DNA lesion in humans is base damage (∼10 000 lesions per cell per day). Base excision repair (BER) is the major repair pathway for this lesion, with poly-(ADP) ribose polymerase 1 (PARP-1) enzyme being one of its essential repair ingredients. Inhibition of PARP-1 activity leads to persistence of SSBs (common intermediates in BER), which once encountered (in S phase) by a DNA-replication fork would result in fork stalling and potential DSBs. The formation of the latter can be repaired by HR. However, if impaired by BRCA1 or BRCA2 loss could result in cell cycle arrest and/or cell death. This potential synthetic lethal condition was tested and verified by the groups of Thomas Helleday and Alan Ashworth, while using PARP-1 inhibitors and BRCA1- or BRCA2-deficient cells (Bryant *et al*, 2005; Farmer *et al*, 2005). In view of recent discoveries of additional roles for PARP-1 in DNA repair, besides BER, the suggested mechanism for the synthetic lethality between PARP-1 inhibitors and BRCA1/BRCA2 deficiency, based on PARP-1-dependent ablation of BER, is but one of the several potential explanations (see Helleday *et al*, 2008; Lord *et al*, 2008). The homozygote BRCA1- or BRCA2-deficient cells were found to be 100- to 1000-fold more sensitive to PARP-1 inhibitors than the wild-type or heterozygote cell lines. The latter is particularly important, as normal, non-tumour cells in patients bearing *BRCA1/BRCA2* mutations are heterozygotes. This monotherapy approach has a potentially large therapeutic window, with higher specificity and less side effects than standard cytotoxic chemotherapy.

The proven synthetic lethality between deficiencies in the HR-dependent DNA repair genes, *BRCA1* or *BRCA2*, and PARP-1 inhibition has opened up several directions of clinical trials. Several PARP-1 inhibitors are currently in phase I or II of clinical monotherapy trials in *BRCA1/BRCA2*-deficient breast or ovarian cancers (Helleday *et al*, 2008; Lord and Ashworth, 2008).

To augment the DNA-damage burden (on top of the spontaneous DNA damage), which cannot be repaired due to PARP-1 inhibition and deficiency in HR repair (BRCA1/BRCA2 deficiency), combination therapies are designed in which effective chemotherapeutics affecting DNA integrity are added together with PARP-1 inhibitors; this is a good example for the model shown in Figure 1C, in which the outcome is DNA integrity affecting cell survival. Accordingly, early clinical trials are underway in which either one of the alkylating agents temozolomide, carboplatin, mitomycin or other DNA-damaging agents are added in combination with a PARP-1 inhibitor to patients with mutant BRCA1/BRCA2 tumours (see details in Helleday *et al*, 2008 ; Lord and Ashworth, 2008). Also, in view of the fact that a fraction of the triple-negative (ER*α*^−^, PR^−^, HER2^−^) breast carcinomas display BRCA1/BRCA2 deficiency, phase-II clinical trials of this patient group, with PARP-1 inhibitors on their own or together with effective chemotherapeutics, are ongoing (http://clinicaltrials.gov).

## Conclusion

The hopes for generating selective cancer drugs by virtue of their synthetic lethal interactions with tumour-specific aberrations are only beginning to materialise. It seems that early efforts in which model organisms, and yeast in particular, were used for drug discovery, have contributed primarily to methodologies, helping shape our experimental approaches in mammalian systems further on. Seemingly, the ‘hit’ rate in large-scale chemical synthetic lethality screening for anticancer compounds in mammalian systems turned out variable and low, requiring large resources of chemical libraries and automated screening facilities. The greater usage in the academia of HTS centres (such as those supported by the Molecular Libraries and Imaging Initiative and the Initiative for Chemical Genetics) and databases (such as PubChem and ChemBank) should certainly improve the prospects of finding new drug candidates.

The concept of searching for cancer drugs through synthetic lethality seems to be more rewarding when either the assay (FOXO1a in PTEN^−/−^) or the potential target gene (mTOR in PTEN^−/−^) can be decided upon based on data mining and prior knowledge of the normal and tumourigenic biological system. The most advanced case is the PARP-1 inhibitors, on their own or in combination with DNA-damaging chemotherapeutics, which are in phase-II clinical trials with breast/ovarian cancer patients having BRCA1/BRCA2 null tumours. Yet, it should be recalled that PARP-1-inhibitory drugs were readily available since their usage in the nineties as chemosensitisers.

Although the identification of synthetic lethal lead compounds through large-scale chemical screening may be hard to come by because of chemical scarcity and large diversity, genetic synthetic lethality screening for gene target identification through siRNAs (Iorns *et al*, 2007) or constitutive/inducible shRNA libraries (Ngo *et al*, 2006; Schlabach *et al*, 2008, respectively) seems more promising. This is due, in part, to the limited number of human genes, and the increased efficiencies of the RNAi tools. The inclusion, on top of the above, of RNAi screens for sensitisers of cancer drugs (Whitehurst *et al*, 2007), and initiatives such as the ‘Connectivity Map’, should further boost cancer targets and drug identification/validation.

At the same time, it may eventually turn out that the number of synthetic lethal interactions in human tumours/tumour cells is relatively limited, because of cell heterogeneity within tumours and multiplicity of compensatory survival pathways/subpathways, respectively. In this context, because resistance to therapy in many tumours may reside within cancer stem cells/tumour-initiating cells (CSCs/TICs), it will be extremely important to isolate CSCs and perform chemical and genetic synthetic lethality screens within this pure population so that CSC-specific drugs and targets could be identified.
